# Potential of Atoxigenic *Aspergillus flavus* Vegetative Compatibility Groups Associated With Maize and Groundnut in Ghana as Biocontrol Agents for Aflatoxin Management

**DOI:** 10.3389/fmicb.2019.02069

**Published:** 2019-09-06

**Authors:** Daniel Agbetiameh, Alejandro Ortega-Beltran, Richard T. Awuah, Joseph Atehnkeng, Md-Sajedul Islam, Kenneth A. Callicott, Peter J. Cotty, Ranajit Bandyopadhyay

**Affiliations:** ^1^International Institute of Tropical Agriculture, Ibadan, Nigeria; ^2^Department of Crop and Soil Sciences, Kwame Nkrumah University of Science and Technology, Kumasi, Ghana; ^3^Agricultural Research Service, United States Department of Agriculture, Tucson, AZ, United States

**Keywords:** aflatoxin, biocontrol, strain selection, efficacy trials, safer food

## Abstract

Increasing knowledge of the deleterious health and economic impacts of aflatoxin in crop commodities has stimulated global interest in aflatoxin mitigation. Current evidence of the incidence of *Aspergillus flavus* isolates belonging to vegetative compatibility groups (VCGs) lacking the ability to produce aflatoxins (i.e., atoxigenic) in Ghana may lead to the development of an aflatoxin biocontrol strategy to mitigate crop aflatoxin content. In this study, 12 genetically diverse atoxigenic African *A. flavus* VCGs (AAVs) were identified from fungal communities associated with maize and groundnut grown in Ghana. Representative isolates of the 12 AAVs were assessed for their ability to inhibit aflatoxin contamination by an aflatoxin-producing isolate in laboratory assays. Then, the 12 isolates were evaluated for their potential as biocontrol agents for aflatoxin mitigation when included in three experimental products (each containing four atoxigenic isolates). The three experimental products were evaluated in 50 maize and 50 groundnut farmers’ fields across three agroecological zones (AEZs) in Ghana during the 2014 cropping season. In laboratory assays, the atoxigenic isolates reduced aflatoxin biosynthesis by 87–98% compared to grains inoculated with the aflatoxin-producing isolate alone. In field trials, the applied isolates moved to the crops and had higher (*P* < 0.05) frequencies than other *A. flavus* genotypes. In addition, although at lower frequencies, most atoxigenic genotypes were repeatedly found in untreated crops. Aflatoxin levels in treated crops were lower by 70–100% in groundnut and by 50–100% in maize (*P* < 0.05) than in untreated crops. Results from the current study indicate that combined use of appropriate, well-adapted isolates of atoxigenic AAVs as active ingredients of biocontrol products effectively displace aflatoxin producers and in so doing limit aflatoxin contamination. A member each of eight atoxigenic AAVs with superior competitive potential and wide adaptation across AEZs were selected for further field efficacy trials in Ghana. A major criterion for selection was the atoxigenic isolate’s ability to colonize soils and grains after release in crop field soils. Use of isolates belonging to atoxigenic AAVs in biocontrol management strategies has the potential to improve food safety, productivity, and income opportunities for smallholder farmers in Ghana.

## Introduction

Following its discovery nearly 60 years ago, aflatoxin contamination of key staple, economically important crops has attracted global attention (Wu, 2015). Developed nations have stringent aflatoxin standards for food/feed crops, milk, and their derived products (Cheli et al., 2014). This allows protecting consumers from health risks associated with aflatoxin exposure (JECFA, 2018). Aflatoxin contamination not only threatens public health but also curtails trade and economic opportunities from farm enterprises when crops exceed tolerance thresholds (Dzirasah, 2015; Kraemer et al., 2016). In contrast, although aflatoxin standards exist in many developing countries such as Ghana (GSA, 2001, 2013), these are poorly enforced. Maize and groundnut in Ghana are prone to aflatoxin contamination. A recent study mirrored the high prevalence of aflatoxin contamination reported frequently over 50 years with concentrations, in most cases, far exceeding the 15 and 10 ppb acceptable threshold for maize and groundnut, respectively, set by the Ghana Standards Authority (Agbetiameh et al., 2018). The two crops constitute major staple and cash crops for millions with per-capita consumption of 44 (US$ 15) and 12 kg (US$ 25) per annum for maize and groundnut, respectively (MoFA, 2011). Consequently, aflatoxin exposure is common and widespread across Ghana. Exposure begins in the unborn child in the uterus and throughout life (Lamplugh et al., 1988; Kumi et al., 2015). Several studies have documented the myriad of health problems associated with aflatoxins in Ghanaians (Shuaib et al., 2010; Jolly et al., 2013; Afum et al., 2016; UNICEF, 2017).

Aflatoxins are produced by fungi belonging to *Aspergillus* section Flavi (Frisvad et al., 2019). *A. flavus*, the most common aflatoxin-producing species worldwide (Klich, 2007), can be subdivided into two distinct morphotypes, the L and S morphotypes (Cotty, 1989). The S morphotype produces numerous small sclerotia (avg. dia <400 μm), few conidia, and consistently high B aflatoxin levels (Cotty, 1989). The L morphotype produces fewer, larger sclerotia (avg. dia >400 μm), numerous conidia, and variable levels of B aflatoxins. There are L morphotype genotypes that lack the ability to produce aflatoxins (i.e., atoxigenic) due to deletions, inversions, or defects in one or more of the aflatoxin biosynthesis genes (Adhikari et al., 2016). *Aspergillus* fungi can be further subdivided into vegetative compatibility groups (VCGs). Members of a VCG descend from the same clonal lineage and therefore are isolated subpopulations (Leslie, 1993; Grubisha and Cotty, 2010, 2015). Diversity among VCGs can be assessed using simple sequence repeat (SSR) markers. Closely related SSR haplotypes in most cases belong to the same VCG (Grubisha and Cotty, 2010, 2015).

Across the globe, several lineages resembling the *A. flavus* S morphotype have been detected with some of them producing copious amounts of both B and G aflatoxins (Probst et al., 2014; Singh and Cotty, 2019). In West Africa, fungi with S morphotype producing both B and G aflatoxins were known as unnamed taxon S_BG_ (Cardwell and Cotty, 2002; Atehnkeng et al., 2008; Donner et al., 2009; Probst et al., 2014). Unknown taxon S_BG_ fungi may be any of the recently described species *A. aflatoxiformans*, *A. austwickii*, *A. cerealis*, or *A. minisclerotigenes* (Pildain et al., 2008; Frisvad et al., 2019). Here we refer as S_BG_ strains to all fungi with S morphotype producing both B and G aflatoxins.

Interactions between atoxigenic and aflatoxin-producing fungi are complex and coupled with other factors determine the extent of crop aflatoxin content (Cotty and Jaime-Garcia, 2007; Mehl et al., 2012; Atehnkeng et al., 2016). In regions where atoxigenic *A. flavus* have been detected, such genotypes have become valuable active ingredients in biocontrol formulations to mitigate crop contamination (Cotty et al., 2007; Atehnkeng et al., 2008; Abbas et al., 2011; Probst et al., 2011; Tran-Dinh et al., 2014; Mauro et al., 2015; Zhou et al., 2015; Alanis Zanon et al., 2016; Bandyopadhyay et al., 2016). Displacement of toxigenic fungi from the crop environment by the deployment of carefully selected atoxigenic *A. flavus* genotypes results in drastic aflatoxin reductions. This has been demonstrated in various crops grown commercially in the United States, Nigeria, Kenya, Senegal, The Gambia, and Italy (Cotty et al., 2007; Dorner, 2010; Doster et al., 2014; Bandyopadhyay et al., 2016; Mauro et al., 2018). This intervention is highly cost-effective in reducing aflatoxin contamination, curtailing aflatoxin-related diseases, and increasing access to local and international premium markets (Wu and Khlangwiset, 2010; Mehl et al., 2012).

In Ghana, aflatoxin management techniques have focused largely on traditional postharvest interventions (Florkowski and Kolavalli, 2013) and more recently on hermetically sealed bags (Paudyal et al., 2017; Danso et al., 2019). In many cases, postharvest technologies are insufficient in curtailing aflatoxin content to safe levels because crop infection and contamination often begins in the field (Mahuku et al., 2019). Once crops become contaminated, aflatoxins cannot be completely removed (Grenier et al., 2014). The aflatoxin biocontrol strategy that targets the source of infection and contamination, the aflatoxin-producing fungi, has not been developed for the farming system in Ghana. However, several atoxigenic *A. flavus* isolates are associated with both maize and groundnut grown across diverse agroecological zones (AEZs) in Ghana (Agbetiameh et al., 2018). The potential of atoxigenic isolates native to Ghana to competitively displace aflatoxin producers and limit crop aflatoxin content has not been investigated.

Atoxigenic biocontrol products are applied during crop development in a formulation (e.g., sterile wheat, sorghum, barley) that gives the active ingredient fungi reproductive advantages over the fungi naturally residing in the treated soils (Mehl et al., 2012). Spores of the beneficial fungi reproduce on the grain, colonize other organic matter substrates in the field, and then become associated with the treated crop during its development (Mehl et al., 2012; Bandyopadhyay et al., 2016). Criteria to select atoxigenic biocontrol agents include wide distribution of the atoxigenic AAV to which they belong over the target nation and superior ability to limit aflatoxin contamination when challenged with highly toxigenic genotypes (Probst et al., 2011; Atehnkeng et al., 2016). It is also necessary to select genotypes with superior abilities to both out-compete other fungi while in the soil and to efficiently move to the crop to provide the intended protection.

The objectives of this study were to: (i) evaluate 12 native atoxigenic *A. flavus* isolates belonging to genetically diverse atoxigenic AAVs for their abilities to reduce aflatoxin production in laboratory assays; (ii) assess comparative abilities of the 12 isolates to establish in soil and crop (maize and groundnut) niches across three AEZs; (iii) determine the extent of aflatoxin reduction by experimental biocontrol products constituted with the candidate isolates; and (iv) select isolates of superior atoxigenic AAVs for use as active ingredients in biocontrol formulations for crop aflatoxin mitigation in Ghana. Native, ecologically adapted atoxigenic AAVs with wide distribution across several AEZs, and with potential as biocontrol agents were detected. Ability to disperse from soil and establish in grains in the field as an ecological criterion for selection of biocontrol active ingredients is a novelty of this study. The identified atoxigenic AAVs are biological resources that can be used to formulate biocontrol products for aflatoxin mitigation. Use of the representative isolates of the selected AAVs may allow for enhanced crop value and food safety and reduce aflatoxin exposure in humans and livestock.

## Materials and Methods

### Microsatellite Genotyping

In a previous study, 4,736 *A. flavus* L morphotype isolates were examined for their aflatoxin-production potential and it was found that 847 isolates lacked aflatoxin-producing abilities (Agbetiameh et al., 2018). We characterized the 847 atoxigenic isolates using SSR markers developed for *A. flavus* (Grubisha and Cotty, 2009). DNA extraction, multiplex-PCR, and microsatellite genotyping were conducted following previously described protocols (Grubisha and Cotty, 2009, 2010; Callicott and Cotty, 2015; Islam et al., 2018). Over 20% of isolates were subjected to at least three independent PCR and genotyping assays for all loci. This allowed to assess consistency of the data.

### Population Genetic Analyses

**FIGURE 1:**
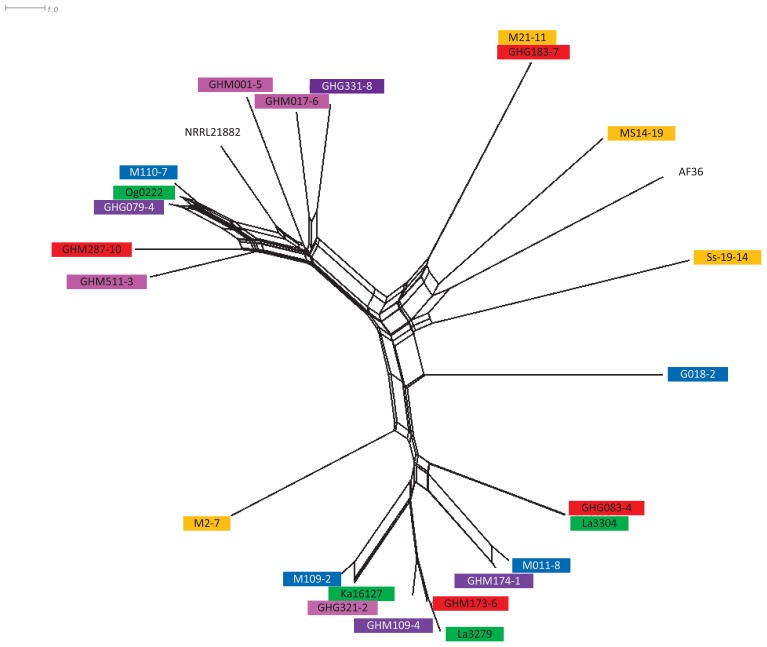
NeighborNet splitstree of 12 selected atoxigenic *Aspergillus flavus* haplotypes from Ghana with other active ingredients of registered aflatoxin biological control products in West Africa. La3279, La3304, Ka16127, and Og0222 are the active ingredients of Aflasafe^TM^ (used in Nigeria, in green); G018-2, M011-8, M109-2, and M110-7 are the active ingredients of Aflasafe BF01 (used in Burkina Faso, in blue); M2-7, M21-11, MS14-19, and Ss19-14 are the active ingredients of Aflasafe SN01 (used in Senegal and The Gambia, in orange); AF36 is the active ingredient of AF36 Prevail^TM^; and NRRL21882 is the active ingredient of Afla-Guard^TM^ (both registered for use in the United States) (Ortega-Beltran and Bandyopadhyay, 2019). Isolates composing experimental product A are in purple, isolates composing experimental product B are in red, and isolates composing experimental product C are in pink. Length of branches are proportional to distances between isolates.

After genotyping, isolates were manually assigned to haplotypes defined by identity across 17 SSR markers (Grubisha and Cotty, 2009). Haplotype frequency was calculated following sample correction, such that a haplotype was only counted once per individual sample. Frequencies were then calculated on a per sample basis (data not shown). Twelve atoxigenic isolates were chosen (Table 1) for testing based on a combination of per sample haplotype frequency, presence in other West African countries, and similarity to atoxigenic biocontrol active ingredients already in use in other West African countries (Figure 1). Frequently encountered haplotypes were assumed to be already well adapted to Ghana. Isolates belonging to AAVs already selected as active ingredients of biocontrol products have a known ability to reduce aflatoxins when properly applied to crops.

**TABLE 1:** Origin of a toxigenic isolate and one atoxigenic isolate each of 12 haplotypes of *Aspergillus flavus* used in the current study.

**Isolate name^a^**	**Crop**	**AEZ^b^**	**Location^c^**	**Community^d^**	**Incidence^e^**
GHM001-5	Maize	DS	Nsawam-Adoagyiri	Nsawam	11
GHM017-6	Maize	HF	Ejisu-Juaben	Hwereso	22
GHG079-4	Groundnut	DS	Atebubu-Amantin	Ahotokrom	5
GHG083-4	Groundnut	DS	Atebubu-Amantin	Ahotokrom	5
GHM109-4	Maize	HF	Ejura-Sekyedumase	Teacher Krom	2
GHM173-6	Maize	HF	Wenchi	Nyamebekyere	6
GHM174-1	Maize	HF	Wenchi	Nyamebekyere	14
GHG183-7	Groundnut	DS	Bole	Carpenter	2
GHM287-10	Maize	SGS	Wa West	Varempere	8
GHG321-2	Groundnut	SGS	Nabdam	Asonge	2
GHG331-8	Groundnut	SGS	Talensi	Pwalugu	10
GHM511-3	Maize	DS	Central Tongu	Bakpa-Ajane	14
GHG040-1^f^	Groundnut	HF	Mampong	Sataso	–

*^*a*^Each isolate belonged to a distinct haplotype which corresponded to a unique African *Aspergillus flavus* vegetative compatibility group. Haplotype refers to multilocus haploid genotypes based on allele calls at each of 17 SSR loci (Grubisha and Cotty, 2009; Table 2). ^*b*^AEZ, agro-ecological zones; DS, derived savanna; HF, humid forest; SGS, southern Guinea Savanna. ^*c*^Administrative district where a community is located. ^*d*^Name of community where household from which maize or groundnut sample containing atoxigenic/toxigenic isolate was found. ^*e*^Number of isolates with similar haplotype encountered among the 847 isolates genotyped. ^*f*^GHG040-1 is an aflatoxin-producing *A. flavus* isolate. All others are atoxigenic genotypes.*

Simple sequence repeat data were re-coded from amplicon size to the number of repeats prior to assessing genetic relationships among all haplotypes. Phylogenetic relationships among the 12 selected genotypes and other registered biocontrol genotypes were assessed with Genodive (Meirmans and Van Tienderen, 2004) after which SplitsTree 4.14.6 (Huson and Bryant, 2005) was used to create a NeighborNet tree (Figure 1).

### Atoxigenic *Aspergillus flavus* L Morphotype Isolates

The population genetic analyses revealed 12 dominant atoxigenic SSR haplotypes widely distributed across different locations of Ghana (Table 1). The origin and distribution of atoxigenic and aflatoxin-producing genotypes is summarized in Table 1. Tester pairs of VCGs were developed for 11 of the 12 SSR haplotype groups following previously described protocols (Cove, 1976; Bayman and Cotty, 1991). It was not possible to obtain a complementary pair of *nit* auxotrophs for isolate GHG183-7. The concordance between SSR haplotype and VCG for 11 of the 12 groups was then tested using vegetative compatibility analyses. These VCGs were termed as AAVs.

### Laboratory Competition Assays

Representative isolates of the 12 SSR haplotypes were evaluated for their ability to limit aflatoxin accumulation when challenged with *A. flavus* isolate GHG040-1, a potent aflatoxin producer native to Ghana, in laboratory competition assays as described by Probst et al. (2011).

To prepare inocula, single-spored isolates, maintained for long-term storage on silica grains, were grown on 5–2 agar [(5% V-8 juice (Campbell Soup Company, Camden, NJ, United States), 2% Bacto-agar (Difco Laboratories Inc., Detroit, MI, United States), pH 6.0)] at 31°C for 7 days (Cotty, 1989). Spore suspensions of each isolate were prepared in 0.1% TWEEN 80^®^ and adjusted to 10^6^ spores ml^–1^ using a turbidimeter (Atehnkeng et al., 2014). A 1-ml spore suspension of the individual atoxigenic isolates and the aflatoxin producer, and mixtures of each atoxigenic/aflatoxin-producing isolate (ratio = 1:1) were separately inoculated on 10 g of autoclaved maize grains. Maize inoculated with 1-ml sterile distilled water served as negative control. Inoculated grains, five replications per treatment, were incubated for 7 days (31°C, dark). The experiment was conducted twice (test 1 and test 2). In test 1, all except atoxigenic isolate GHG083-4 was evaluated.

Following incubation, aflatoxins were extracted from maize fermentations as previously described (Agbetiameh et al., 2018). Briefly, fermentations were combined with 50 ml 70% methanol. Suspensions were shaken on a Roto-Shake Genie (Scientific Industries, Bohemia, NY, United States) for 30 min at 400 rpm and filtered through Whatman No. 1 filter paper (Whatman International Ltd., Maidstone, United Kingdom). Filtrates were collected in 250 ml separatory funnels, combined with 5 ml distilled water, and extracted with 15 ml methylene chloride. The methylene chloride phase was filtered through a bed of 25 g anhydrous sodium sulfate contained in fluted Whatman No. 4 filter paper, combined, and evaporated to dryness in a fume hood (Cotty and Cardwell, 1999). Residues were dissolved in 1 ml methylene chloride, spotted (4 μl) alongside aflatoxin standards (Supelco, Bellefonte, PA, United States) on thin layer chromatography (TLC) Aluminum (20 cm × 10 cm) Silica gel 60 F_254_ plates (Merck, Darmstadt, Germany) and developed with diethyl ether–methanol–water (96:3:1) (Probst and Cotty, 2012). Aflatoxins were quantified directly on TLC plates with a scanning densitometer (CAMAG TLC Scanner 3) and quantification software (winCATS 1.4.2, Camag, AG, Muttenz, Switzerland) (Agbetiameh et al., 2018).

### Formulation of Experimental Biocontrol Products

Three experimental biocontrol products (named A, B, and C) were composed each with four representative atoxigenic isolates of different haplotypes and manufactured in Ibadan, Nigeria (Table 2). To prepare each product, spores of the four atoxigenic isolates were obtained from 5-day-old cultures grown on 5–2 agar to prepare inoculum in bulk. Spores were dislodged and suspended in 0.1% TWEEN 80^®^ and adjusted to 10^6^ spores ml^–1^ as above. Spores of each atoxigenic isolate were independently reproduced in glass bottles containing sterilized sorghum grain as follows. Prior to inoculation, sorghum grain was pre-conditioned in sterile 1-L plastic bottles. Moisture content of sorghum grain was increased to 30% by adding sterile distilled water and bottles were rolled for 4 h on a 240 Vac Benchtop Roller (Wheaton, Millville, NJ, United States). Thirty grams of pre-conditioned grain were added to 250-ml glass bottles along with two Teflon balls (1/2″ diameter) and autoclaved (20 min, 121°C). Each cooled bottle containing sorghum was independently inoculated with 4 ml of spore suspension of each atoxigenic isolate. After incubation (7 days, 31°C), 125 ml sterile 0.1% TWEEN^®^ 20 was added to each bottle to harvest spores. Bottles were placed on a Roto-Shake Genie reciprocal shaker (Scientific Industries, Bohemia, NY, United States) at 200 rpm for 20 min. The Teflon balls facilitated dislodging spores from sorghum grains. For each atoxigenic strain, a suspension was adjusted to 4 × 10^7^ spores ml^–1^ as above. To prepare 100 kg of each experimental product, a spore suspension (1 l, 4 × 10^7^ spores ml^–1^) of the constituent atoxigenic genotypes was individually combined with 150 ml of a polymer (Sentry^TM^, Precision Laboratories, Waukegan, IL, United States) and 200 ml of a blue non-toxic dye (Prism^TM^, Milliken and Company, Spartanburg, SC, United States) and coated on roasted, sterile sorghum grain with a seed treater (Bandyopadhyay et al., 2016). Following phytosanitary certification by the Nigeria Plant Quarantine Service and the issuance of import permit by the Plant Protection and Regulatory Services Directorate (PPRSD) of Ghana’s Ministry of Food and Agriculture (MoFA), the three experimental products were transported to Ghana for evaluation in farmer field trials.

**TABLE 2:** Composition of experimental aflatoxin biocontrol products, each containing a mixture of four atoxigenic *Aspergillus flavus* vegetative compatibility groups represented by their type isolates.

**Product**	**Isolate**	**SSR locus**																
		
		**AF28**	**AF13**	**AF43**	**AF22**	**AF31**	**AF53**	**AF34**	**AF42**	**AF8**	**AF16**	**AF54**	**AF17**	**AF11**	**AF66**	**AF64**	**AF63**	**AF55**
A	GHM174-1	113	145	390	192	349	134	301	159	171	169	161	359	141	261	169	127	180
	GHG331-8	119	148	379	144	312	131	296	146	166	169	161	385	126	269	163	127	172
	GHG079-4	119	128	379	144	312	131	296	150	166	169	161	353	132	269	161	127	180
	GHM109-4	135	145	385	192	346	134	301	181	171	169	161	356	141	261	169	127	178
B	GHM173-6	135	145	385	192	346	134	301	181	189	169	161	356	141	261	169	127	180
	GHG083-4	131	135	385	192	315	134	323	159	171	169	161	359	141	255	169	127	184
	GHM287-10	119	141	399	144	312	131	298	150	166	169	161	368	135	271	161	127	180
	GHG183-7	119	145	411	188	325	144	314	168	180	175	172	353	150	269	195	127	172
C	GHM017-6	119	145	426	144	312	131	296	146	174	169	161	374	129	269	163	127	178
	GHM511-3	119	128	399	144	312	131	296	150	174	169	161	368	132	269	159	127	174
	GHG321-2	135	145	385	192	367	134	301	159	160	169	161	362	141	261	169	127	184
	GHM001-5	119	128	387	144	312	131	296	143	168	169	161	374	138	271	161	125	178

*For each isolate, the amplicon size at each SSR locus is given.*

### Field Sites, Plots, and Trial Establishment

Field trials were conducted in 2014 during the major cropping season in Northern Ghana and minor season in the Middle Belt. The trials were conducted in five regions located in three AEZs. In each region, the fields were distributed in two districts. The two cropping seasons and the AEZs’ characteristics have been described previously (Agbetiameh et al., 2018). Farmers and their field selection was done in collaboration with Agricultural Extension Agents from the Department of Agriculture of MoFA in the respective districts following stakeholder sensitization and training workshops. In each district, five maize and five groundnut fields (size ≥ 2 ha) were selected. Farmers grew their crops according to their own agronomic practices. Each field was divided into four equal-sized plots separated by 5 m from each other. Assignment of plots to treatments across field locations was done using a randomized complete block design (RCBD). Three plots within a block were assigned treatment to one of the three experimental products. The remaining plot was left untreated and served as control. In each district, treatments were replicated five times. When field sizes were <2 ha (mostly groundnut fields), individual fields in a group of four nearby fields were considered as plots. Experimental products were broadcasted by hand (10 kg ha^–1^) to field soils 2 weeks before flowering and following weeding and/or fertilizer application by farmers. From each plot, before product application and also at harvest, soil samples (up to 2.5 cm depth) were taken randomly from at least 15 different spots resulting in a composite sample of about 150 g (Atehnkeng et al., 2014). Grain samples comprising 25 maize ears and approximately 1-kg groundnut (in-shell) were collected at harvest.

### Analysis of *Aspergillus* Section *Flavi* in Soils and Grains

Soil samples were dried in a forced-air oven (50°C, 48 h). Samples with clods were pulverized and sieved through 2 mm wire mesh to remove gravel and large particles. Grains were manually shelled, and 500 g were milled using a laboratory blender (Waring Commercial, Springfield, MO, United States) for 1 min in a 250 ml stainless steel blending jar (MC-2). Milled samples were stored at 4°C before aflatoxin and microbial analyses. The blending jar was washed between samples with 80% ethanol to prevent microbial and aflatoxin cross contamination. *Aspergillus* section *Flavi* fungi in soil and grains were isolated using dilution plate technique on modified rose Bengal Agar as described previously (Atehnkeng et al., 2014). Plates were incubated for 3 days (31°C, dark). From each sample, 12 discrete *Aspergillus* species colonies were sub-cultured on 5–2 agar (31°C, 7 days) and then assigned to their corresponding species based on macroscopic and microscopic characteristics (Pitt and Hocking, 2009). Sporulating cultures of each isolate were saved as agar plugs in 4 ml vials containing 2 ml sterile distilled water until further characterization.

### Aflatoxin Determination in Grain Samples

Aflatoxin levels in maize and groundnut sampled at harvest were examined to determine the extent of contamination in grains from treated and control plots. Aflatoxins were extracted from maize by combining 20 g ground sample with 100 ml of 70% methanol (Atehnkeng et al., 2008). For groundnut, 20 g of ground sample was combined with 100 ml of 80% methanol (Cole and Dorner, 1993). Aflatoxins were extracted, combined, separated on TLC plates, and quantified as described above.

### Incidence of Atoxigenic Genotypes

Frequencies of *A. flavus* belonging to the applied AAVs of the three experimental products were examined in soils and grains. Nitrate non-utilizing (*nit*) auxotrophs were generated for all recovered *A*. *flavus* L morphotype isolates (Grubisha and Cotty, 2010). Briefly, a spore suspension of each isolate (approximately 1,000 spores in 15 μl) was seeded into a well at the center of a plate containing mutant selection medium (Czapek-dox broth, 25 g l^–1^ KClO_3_, 10 ml l^–1^ rose Bengal, 2% Bacto-agar, pH 7.0). Seeded plates were incubated at 31°C for 7–30 days. Spontaneous auxotrophic sectors were transferred to a purification medium (Czapek-dox broth, 15 g l^–1^ KClO_3_, 2% Bacto-agar, pH 6.5) for 3 days to clean up and stabilize *nit* mutants. A mutant sector was subsequently transferred onto 5–2 agar, and incubated for 5 days at 31°C. Plugs of sporulating mutants were stored in 4 ml glass vials containing 2 ml sterile distilled water for use in complementation assays. Assignment of mutants of isolates to an AAV was based on pairing the isolate auxotroph with complementary tester auxotrophs of each applied AAV (Grubisha and Cotty, 2010). A single complementation test was performed on starch agar (36 g l^–1^ dextrose, 3 g l^–1^ NaNO_3_, 2% Bacto-agar, 2% soluble starch, pH 6.0) (Cotty and Taylor, 2003) where three wells (3 mm dia, 1 cm apart) were made in a triangular pattern at the center of the plate. Two wells were each seeded with 15 μl of either of the tester pair while the third well was seeded with the isolate auxotroph being characterized. Plates were incubated for 5–10 days at 31°C. Auxotrophs forming a stable heterokaryon with one or both tester auxotrophs of an applied AAV were assigned to that AAV and were considered to be the applied genotype. In all, a total of 47,520 vegetative compatibility tests were conducted.

### Data Analysis

All statistical tests were performed with SAS (version 9.4, SAS Institute Inc., Cary, NC, United States). Prior to data analysis, all response variables were log-transformed to stabilize variances. Means of the response variables were subjected to analysis of variance (ANOVA) and separated with Fisher’s protected least significant difference (LSD) test (α = 0.05). Pairwise comparison means of response variables from treated and control plots were conducted using Student’s *t*-test (α = 0.05). Applied AAVs were ranked separately by their incidence in soil and grain samples across different geographical locations. To calculate the rank, the proportion of the number of (i) AEZ (*n* = 3), (ii) regions (*n* = 5), (iii) districts (*n* = 10), and (iv) samples (*n* = 30) where the AAV was detected and (v) the proportion of isolates of the AAV detected (*n* = 360) was summed. Higher the sum, higher (1 = highest, 11 = lowest) the rank. For example, AAV GHM287-10 in maize was detected in the 3 AEZ (3/3 = 1.0), 5 regions (5/5 = 1.0), 8 districts (8/10 = 0.8), 17 samples (17/30 = 0.57), and 75 isolates were detected (75/360 = 0.21) for a total of 3.58.

## Results

### Identification of Dominant Atoxigenic Genotypes

Out of the 847 atoxigenic *A. flavus* L morphotype isolates identified previously (Agbetiameh et al., 2018), there were 454 unique and diverse haplotypes. Among those haplotypes, 12 were widely distributed across Ghana (Table 1) but not closely related (Figure 1). AAV grouping of 11 of the 12 groups concurred with the grouping revealed by SSRs (data not shown). Mutants of isolate GHG183-7 did not complement with tester pairs of any of the 11 AAVs. Therefore, GHG183-7 was considered another AAV.

The SSR signatures for identifying the representative isolates of AAVs constituting the experimental products are reported in Table 2. None of the locus was monomorphic among the examined isolates. The number of alleles per locus ranged from 2 to 7 (Table 2).

### Aflatoxin Inhibition Potential of Atoxigenic Genotypes in Competition Tests

When inoculated individually, none of the 12 atoxigenic isolates produced aflatoxins on maize grains (LOD = 0.1 μg kg^–1^), as in the previous study (Agbetiameh et al., 2018). The aflatoxin-producing isolate GHG040-1 produced high aflatoxin B_1_ levels (>51.0 mg kg^–1^) on maize grains in both tests, as expected. Marked variations (*P* < 0.01) were detected in the aflatoxin inhibition potential of atoxigenic isolates when co-inoculated with the aflatoxin producer. Aflatoxin reductions ranged from 92.8 to 98.7% (Table 3). In test 1, atoxigenic isolates GHM173-6 and GHM511-3 significantly (*P* < 0.0001) reduced aflatoxin accumulation by the aflatoxin producer to <1.0 mg kg^–1^, the lowest level among all combinations. GHG183-7 had the least aflatoxin inhibition potential (5.59 mg kg^–1^). However, that level was also significantly (*P* < 0.0001) lower than in grains inoculated solely with the aflatoxin producer. GHG083-4 was not selected when test 1 was conducted, hence no aflatoxin inhibition data were generated in test 1 (Table 3).

**TABLE 3:** Aflatoxin B (B_1_ + B_2_) content of maize in μg/kg during co-inoculation of atoxigenic isolates and an aflatoxin-producer.

**Isolate**	**Test 1**		**Test 2**	
		
	**Aflatoxin B (mg kg^–1^)^a^**	**Reduction (%)^b^**	**Aflatoxin B (mg kg^–1^)^a^**	**Reduction (%)^b^**
GHM001-5	1.22ab	98.4	2.51abc	95.1
GHM017-6	2.81d	96.4	2.86abc	94.4
GHG079-4	1.49ab	98.1	1.61ab	96.8
GHG083-4^c^	–	–	4.77cd	90.6
GHM109-4	1.55ab	98.0	1.32ab	97.4
GHM173-6	0.98a	98.7	1.23ab	97.6
GHM174-1	1.83bc	97.6	0.90a	98.2
GHG183-7	5.59f	92.8	6.47d	87.3
GHM287-10	1.57ab	97.9	1.34ab	97.4
GHG321-2	2.59d	96.7	2.85abc	94.4
GHG331-8	4.52e	94.2	3.35bc	93.4
GHM511-3	0.99a	98.7	2.66abc	94.8
GHG040-1^d^	77.56	–	51.05	–

*^*a*^Aflatoxin B values having a common letter are not significantly different according to Fischer’s Least Significant Difference (LSD) test (α = 0.05). ^*b*^Percent aflatoxin B reduction was calculated as [1 – (aflatoxin B content in maize co-inoculated with both toxigenic and atoxigenic isolate/aflatoxin B content in maize inoculated with the aflatoxin-producing isolate alone)] × 100. ^*c*^No data were generated for the atoxigenic isolate GHG083-4 in the first test. ^*d*^GHG040-1 is an aflatoxin-producing isolate.*

Similar results were observed in test 2. Aflatoxin reductions ranged from 87.3 to 98.2% (Table 3). The lowest toxin inhibition (6.47 mg kg^–1^) was by GHG183-7, as in test 1. GHM174-1 reduced aflatoxin the most (0.90 mg kg^–1^).

### Quality Control of the Experimental Products

All carrier grains of all batches of the experimental products were colonized only by *A. flavus*. Other microorganisms were not recovered in any of the grains. The recovered *A. flavus* fungi were solely composed of the active ingredient AAVs composing the experimental products. Other AAVs of *A. flavus* were not detected in any of the batches. In each experimental product, each of the four active ingredient AAVs was found on 25 ± 3% carrier grains of the examined batches. Each gram of product contained, on average, 3500 ± 300 colony forming units (CFUs) of the active ingredient fungi.

### Aflatoxin Concentration in Crop Samples

**FIGURE 2:**
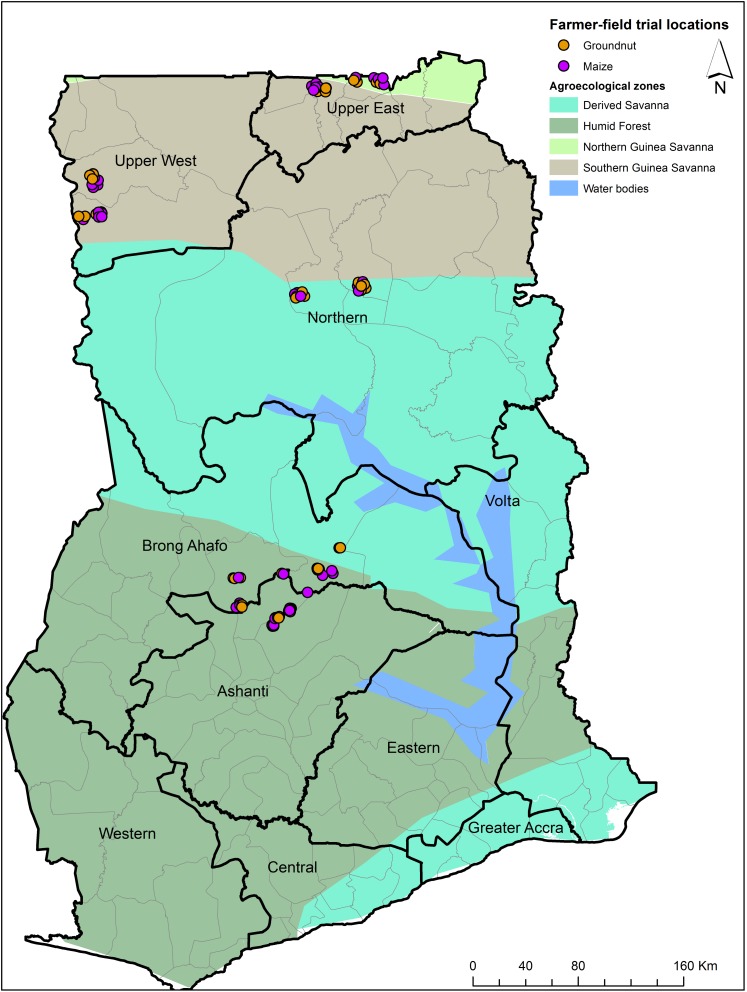
Map of Ghana indicating locations where field trials were conducted in maize and groundnut during 2014.

Field trials were conducted in 2014 in 10 districts from five regions located in three AEZs in Ghana (Figure 2). Across all AEZs, substantially (*P* < 0.05) less aflatoxins accumulated in grains from plots treated with the experimental products, compared to untreated grains. Treated groundnut contained 70.5–99.7% less aflatoxins than those untreated. Across AEZs, aflatoxin levels in treated groundnut ranged from 1 to 61 μg kg^–1^ with those from humid forest (HF) containing safe levels. Aflatoxin content in untreated groundnut ranged from 58 to 302 μg kg^–1^ (Table 4). In maize, up to 100% reduction was detected in treated crops. Aflatoxin concentration was below 0.1 μg kg^–1^ in treated maize while it ranged from 0.8 to 7.8 μg kg^–1^ in control plots (Table 4).

**TABLE 4:** Aflatoxin content (μg kg^–1^) in groundnut and maize kernels at harvest from treated and control fields across three agroecological zones (AEZs) in Ghana during 2014 cropping season.

**AEZ^a^**	***N*^b^**	**Treatment^c^**	**Aflatoxin concentration (μg kg^–1^)**			
			
			**Groundnut**		**Maize**	
				
			**Mean**	**Reduction (%)^d^**	**Mean**	**Reduction (%)^d^**
DS	9	A	40.2	86.7	0	100
	9	B	0.9	99.7	0	100
	9	C	1.5	99.5	0.4	50.0
	9	Control	302.0		0.8	
HF	9	A	8.3	85.7	0	100
	9	B	3.2	94.5	0.4	94.9
	9	C	0.4	99.3	0.2	97.4
	9	Control	57.9		7.8	
SGS	12	A	45.6	78.1	0.1	96.6
	12	B	13.7	93.4	0.2	93.1
	12	C	61.3	70.5	0.1	96.6
	12	Control	208.0		2.9	

*^*a*^DS, derived savanna; HF, humid forest; SGS, southern guinea savanna. ^*b*^Number of treated and control plots. ^*c*^Experimental products used in the current study (Table 2). Each experimental product (A, B, and C) contained four atoxigenic isolates each representing a unique atoxigenic African *Aspergillus flavus* vegetative compatibility group. Control refers to plots to which no experimental product was applied. ^*d*^Percent aflatoxin reduction = [1 – (mean aflatoxin content of grains from treated plots/mean aflatoxin content of grains from control plots)] × 100.*

### *Aspergillus* Fungal Communities in Soils and Grains

Four main members within *Aspergillus* section *Flavi* (*A. flavus* L morphotype, S_BG_ strains, *A. parasiticus*, and *A. tamarii*) were recovered from soil before application and at harvest, and on grain collected at harvest. In all substrates, *A. flavus* L morphotype dominated the communities with frequencies greater than 83% (Table 5). Prior to application of experimental products, incidence of L morphotype in field soils ranged from 87.7% in HF to 99.1% in derived savannah (DS). Frequencies of S_BG_ strains, *A. parasiticus*, and *A. tamarii* were low (range = 0–9.9%; Table 5).

**TABLE 5:** Community structure of *Aspergillus* section *Flavi* in soils and maize samples from control and treated plots across three agroecological zones (AEZs) in Ghana.

**AEZ^a^**	**Treatment^b^**	***N*^c^**	***Aspergillus* species/strain distribution^d^ (%)**											
			
			**Soil before application**				**Soil at harvest**				**Grain**			
					
			***L***	***S*_BG_**	***P***	***T***	***L***	***S*_BG_**	***P***	***T***	***L***	***S*_BG_**	***P***	***T***
DS	A	9	97.8	2.2	0	0	100^∗^	0^∗^	0	0	100	0	0	0
	B	9	93.4	3.2	0.8	2.6	99.3^∗^	0.7^∗^	0	0	100	0	0	0
	C	9	99.1	0.9	0	0	99.3^∗^	0.7^∗^	0	0	100	0	0	0
	Control	9	98.4	1.6	0	0	82.7	17.3	0	0	98.6	1.4	0	0
HF	A	9	91.4	2.8	2.9	2.9	97.9^∗^	1.4^∗^	0	0.7	100	0	0	0
	B	9	87.7	9.9	1.5	0.9	99.3^∗^	0.7^∗^	0	0	100	0	0	0
	C	9	94.1	1.6	1.3	3.0	97.2^∗^	2.1	0.7	0	100	0	0	0
	Control	9	90.1	7.7	0	2.2	88.2	8.4	1.4	2	99.3	0.7	0	0
SGS	A	12	97.4	0.6	1.3	0.7	98.4	1.6	0	0	100	0	0	0
	B	12	95.0	0	3.2	1.8	97.9	1.0	0	1.1	100	0	0	0
	C	12	94.8	0.6	1.7	2.9	97.9	2.1	0	0	100	0	0	0
	Control	12	95.6	0	0.6	3.8	89.6	6.8	0	3.6	100	0	0	0

*^*a*^DS, derived savanna; HF, humid forest; SGS, southern Guinea savanna. ^*b*^Biocontrol experimental products used in the current study (Table 2). Control refers to plots to which no experimental product was applied. ^*c*^Number of plots analyzed. ^*d*^L, *A. flavus* L morphotype; *S*_*BG*_, *S*_*BG*_ strains; P, *A. parasiticus*; T, *A. tamarii*. An asterisk indicates significant difference in strain/species incidence between a treated plot and control plot within an AEZ by Student’s *t*-test (α = 0.05).*

Frequencies of *A. flavus* L morphotype in treated soils at harvest ranged from 97.2% in HF to 100% in DS. Across AEZs, in control plots, relatively lower L morphotype frequencies were detected in soil at harvest compared to soil before application. Significantly (*P* < 0.05) higher L morphotype frequencies were observed across treated plots in both DS and HF. Generally, incidences of S_BG_ strains, *A. parasiticus*, and *A. tamarii* were lower in treated soils at harvest, compared to soil before application of experimental products. At harvest in DS, the proportions of S_BG_ strains were significantly (*P* < 0.05) higher in untreated soils than in treated soils (Table 5). *Aspergillus* communities in treated maize kernels across all three AEZs were entirely composed of the L morphotype. In control maize, the L morphotype dominated and minor frequencies of S_BG_ strains (up to 1.4%) were found (Table 5). A similar trend in frequencies of L morphotype, S_BG_ strains, *A. parasiticus*, and *A. tamarii* was observed in soils from groundnut fields and groundnut kernels, except that communities in treated groundnut, in addition to the L morphotype, harbored minor proportions of S_BG_ strains (up to 0.7%) (Table 6).

**TABLE 6:** Community structure of *Aspergillus* section *Flavi* in soils and groundnut samples from treated and control plots across three agroecological zones (AEZs) in Ghana.

**AEZ^a^**	**Treatment^b^**	***N*^c^**	***Aspergillus* species/strain distribution^d^ (%)**											
			
			**Soil before application**				**Soil at harvest**				**Grain**			
					
			***L***	***S*_BG_**	***P***	***T***	***L***	***S*_BG_**	***P***	***T***	***L***	***S*_BG_**	***P***	***T***
DS	A	9	97.4	0.9	0.9	0.8	99.3	0	0	0.7	100	0	0	0
	B	9	94.3	1.6	0.8	3.3	98.6	1.4	0	0	99.3	0.7	0	0
	C	9	99.1	0.9	0	0	98.6	1.4	0	0	100	0	0	0
	Control	9	98.3	0.9	0	0.8	95.8	3.5	0.7	0	100	0	0	0
HF	A	9	97.0	2.3	0.7^∗^	0	100^∗^	0^∗^	0	0	100	0	0	0
	B	9	89.5	6.7	3.8	0	99.3^∗^	0.7^∗^	0	0	99.3	0.7	0	0
	C	9	95.5	1.6	2.9	0	95.8^∗^	4.2	0	0	99.3	0.7	0	0
	Control	9	93.6	0	6.4	0	75.7	16.7	6.9	0.7	91.0	9.0	0	0
SGS	A	12	98.7	0	1.3	0	99.5	0	0.5	0	100	0	0	0
	B	12	97.5	1.9	0	0.6	99.5	0	0.5	0	99.5	0.5	0	0
	C	12	99.4	0.6	0	0	99.0	0.5	0	0.5	100	0	0	0
	Control	12	96.9	0.7	1.8	0.6	91.2	6.3	1.0	1.5	99.5	0	0	0.5

*^*a*^DS, derived savanna; HF, humid forest; SGS, southern Guinea savanna. ^*b*^Biocontrol experimental product used in the current study (Table 2). Control refers to plots to which no experimental product was applied. ^*c*^Number of plots analyzed. ^*d*^*L*, *A. flavus* L morphotype; *S*_*BG*_, *S*_*BG*_ strains; *P*, *A. parasiticus*; *T*, *A. tamarii*. An asterisk indicates significant difference in strain/species incidence between a treated plot and control plot within an AEZ by Student’s *t*-test (α = 0.05).*

### Incidence of Applied Atoxigenic AAVs in Grains After Treatment

The individual atoxigenic AAVs composing the applied experimental products showed varying abilities to disperse from treated soils and establish in the grain of treated and control plots. Each AAV was assigned a rank based on their incidence across AEZ, regions, districts, samples, and number of AAV individuals detected. For instance, 75 isolates belonging to AAV GHM287-10 were recovered from 17 maize samples from 8 out of 10 districts in all five regions across all three AEZs, thus being the most dominant applied AAV in treated grains (rank = 1, Table 7). The same AAV was also frequently isolated from control grains (rank = 2, in control grains). Barring a few exceptions (e.g., AAV GHM511-3), most AAVs with high post-release incidence in grains from treated plots also had relatively high incidence in control plots. In contrast, AAV GHM173-6 was the least frequently isolated from grains of both treated and control plots (Table 7).

**TABLE 7:** Rankings^a^ of isolates belonging to atoxigenic African *Aspergillus flavus* vegetative compatibility groups (AAVs) in soils and grain from both maize and groundnut plots treated with three experimental products and their corresponding controls in three agroecological zones (AEZs) in Ghana.

**Experimental product**	**Isolate**	**Plot**	**Soil**		**Grain**		**Average**
							
					
			**Maize**	**Groundnut**	**Maize**	**Groundnut**	
A	GHG331-8	Treated	1	9	5	8	5.75
	GHG331-8	Control	9	5	4	8	6.5
	GHG079-4	Treated	3	8	9	10	7.5
	GHG079-4	Control	6	10	7	9	8.0
	GHM109-4	Treated	5	1	4	5	3.75
	GHM109-4	Control	10	9	3	2	6.0
	GHM174-1	Treated	9	3	2	6	5.0
	GHM174-1	Control	11	8	1	5	6.25
B	GHM173-6	Treated	2	10	11	11	8.5
	GHM173-6	Control	1	10	7	9	6.75
	GHG083-4	Treated	4	4	8	1	4.25
	GHG083-4	Control	8	7	5	6	6.5
	GHM287-10	Treated	11	2	1	2	4.0
	GHM287-10	Control	7	3	2	7	4.75
C	GHM017-6	Treated	8	7	7	9	7.75
	GHM017-6	Control	2	4	8	9	5.75
	GHM511-3	Treated	6	6	3	4	4.75
	GHM511-3	Control	3	1	6	1	2.75
	GHG321-2	Treated	7	11	10	7	8.75
	GHG321-2	Control	5	6	8	3	5.5
	GHM001-5	Treated	10	5	6	3	6.0
	GHM001-5	Control	4	2	7	4	4.25

*^*a*^Applied isolates were ranked separately by their incidence in grain samples across different geographical locations. To calculate the rank, the proportion of the number of (i) AEZ (*n* = 3), (ii) regions (*n* = 5), (iii) districts (*n* = 10), and (iv) samples (*n* = 30) where the AAV was detected, and (v) the proportion of isolates of the AAV detected (*n* = 360) was summed. Higher the sum, higher (1 = highest, 11 = lowest) the rank. For example, AAV GHM287-10 in maize was detected in the 3 AEZ (3/3 = 1.0), 5 regions (5/5 = 1.0), 8 districts (8/10 = 0.8), 17 samples (17/30 = 0.57), and 75 isolates were detected (75/360 = 0.21) for a total of 3.58, which was rounded to 4.0.*

Abilities of the applied AAVs to move into groundnut kernels also varied. Generally, incidence of applied AAVs was relatively lower in groundnut than in maize (Table 8). The most prevalent applied AAV was GHG083-4 with 52 member isolates found in 15 samples from all 10 districts in all the regions of the three AEZs. On the other hand, no isolate of AAV GHM173-6 was recovered in groundnut from any field (Table 7).

**TABLE 8:** Selected isolates belonging to atoxigenic African *Aspergillus flavus* vegetative compatibility groups composing two biocontrol products for further evaluation in Ghana.

**S/N**	**Isolate**	**Product**
1.	GHG079-4	Aflasafe GH01
2.	GHG083-4	Aflasafe GH01
3.	GHG321-2	Aflasafe GH01
4.	GHM174-1	Aflasafe GH01
5.	GHM511-3	Aflasafe GH02
6.	GHM109-4	Aflasafe GH02
7.	GMH001-5	Aflasafe GH02
8.	GHM287-10	Aflasafe GH02

There were some AAVs with high ranking positions in both crops. For example GHM287-10 was the 1st and 2nd ranked AAV in treated maize and groundnut, respectively (Table 7). However, also in treated grains, GHG083-4 was the 1st and 8th ranked AAV in groundnut and maize, respectively. Success of establishment of an AAV in one crop was not always associated with success in the other crop.

### Selected Isolates of Atoxigenic AAVs for Aflatoxin Biocontrol in Ghana

Based on incidence of the candidate AAVs in maize and groundnut grains following their release across locations, regions, and AEZs, and SSR data (Table 2), one representative atoxigenic isolate of eight AAVs with widest distribution and with superior ability to reduce aflatoxin contamination in grains were selected as active ingredients of two biocontrol products (Table 8).

## Discussion

In the current study, 12 atoxigenic AAVs native to Ghana were identified and a representative isolate of each AAV were evaluated for their potential as biocontrol agents for aflatoxin mitigation of both maize and groundnut grown across various AEZs. The 12 evaluated isolates successfully inhibited aflatoxin production (range = 87.3–98.7% less) when co-inoculated with a potent aflatoxin-producing *A. flavus* isolate native to Ghana in laboratory tests. Aflatoxin reduction levels were comparable to those detected in elite atoxigenic *A. flavus* isolates endemic to the United States (Cotty and Bayman, 1993; Ortega-Beltran et al., 2019), Nigeria (Atehnkeng et al., 2008), Kenya (Probst et al., 2011), Italy (Mauro et al., 2015), and China (Zhou et al., 2015). In sub-Saharan Africa, specifically Nigeria, Kenya, Senegal, The Gambia, and Burkina Faso, similar evaluations resulted in identification and selection of unique AAVs for the development of atoxigenic products tailored to each country (Atehnkeng et al., 2008; Probst et al., 2011; Bandyopadhyay et al., 2016). To our knowledge, the current work is the first published study of selection of active ingredients of an aflatoxin biocontrol product supported by information on their ability to disperse to crops from a formulated product applied on soil.

For over two decades, atoxigenic aflatoxin biocontrol has been demonstrated as the most effective and sustainable strategy to reduce crop aflatoxin content (Cotty, 1994; Dorner, 2004, 2010; Cotty et al., 2007; Mehl et al., 2012; Atehnkeng et al., 2014; Doster et al., 2014; Grubisha and Cotty, 2015). This strategy is based on the deployment of native atoxigenic isolates of VCGs that both competitively displace aflatoxin-producers and inhibit aflatoxin biosynthesis. Isolates belonging to atoxigenic VCGs locally adapted to specific AEZs and cropping systems, and with superior competitive ability to exclude aflatoxin producers from the target crop or environment are used in aflatoxin management programs (Cotty et al., 2007; Dorner, 2010; Abbas et al., 2011; Mehl et al., 2012; Doster et al., 2014; Bandyopadhyay et al., 2016). In keeping with this paradigm, 12 isolates belonging to genetically diverse SSR haplotypes/AAVs with wide distribution across Ghana (Islam et al., 2015) were identified from 847 atoxigenic isolates described previously (Agbetiameh et al., 2018) using 17 SSR loci (Grubisha and Cotty, 2009).

Mehl et al. (2012) emphasized that VCG analyses provide insights into the diversity of fungal communities including aflatoxin production and inhibition potentials. Indeed, variation in aflatoxin inhibition among representative isolates of the 12 atoxigenic AAVs was expected. GHG183-7 was least effective at inhibiting aflatoxin contamination in laboratory assays. This suggests that GHG183-7 is a poor competitor during host colonization (Mehl and Cotty, 2010) compared to the other evaluated isolates. Atehnkeng et al. (2008) emphasized that reduced competitiveness in laboratory conditions may provide an early signal of low competitiveness during crop development and, subsequently, less efficacy in practice. Furthermore, Atehnkeng et al. (2008) suggested exclusion of atoxigenic isolates with considerably less competitive abilities prior to expensive, time consuming field studies. Apart from being the least competitive isolate, we were unable to obtain a complementary pair of *nit* auxotrophs for this isolate. Whether this isolate is self-incompatible as reported in studies of *Aspergillus* and other genera (Correll et al., 1987; Krnjaja et al., 2013) needs to be clarified. Consequently, frequencies of AAV GHG183-7 were not evaluated even though an isolate of that AAV was a constituent of experimental product B.

Use of native AAVs in biocontrol programs offers better adaptation to target agroecosystems (Probst et al., 2011) and long-term establishment of *A. flavus* communities with low aflatoxin-producing potential (Mehl et al., 2012). Genetic variability among *A. flavus* individuals results in differential adaptation to various agroecological niches (Cotty and Mellon, 2006; Mehl and Cotty, 2013). Indeed, this phenomenon was expected among the 12 atoxigenic isolates evaluated in the current study. Studies of adaptive potentials of these isolates across three AEZs suggest extents of adaptation of their corresponding AAVs to the conditions of the three evaluated AEZs. For instance, the atoxigenic isolate GHM173-6 was the most effective at reducing aflatoxin concentrations in laboratory assays (Table 3) and was also one of the isolates most commonly found in treated and untreated maize soil (Table 7 and Supplementary Table 1). This notwithstanding, GHM173-6 was the least encountered in maize grain from all field locations across regions and was never recovered from groundnut (Table 7 and Supplementary Tables 2–4). On the contrary, GHM511-3 exhibited both high aflatoxin inhibition potential and high recovery on both maize and groundnut across regions and all three AEZs (Tables 3, 7). These observations support both competitiveness and crop adaptation as important criteria for selection of active ingredient AAVs for biocontrol formulations.

A major objective of the field evaluations of multiple isolates was to detect atoxigenic isolates belonging to AAVs with superior ability to establish in the crop after introduction in formulated product on the soil (Table 7). Apart from aflatoxin reduction of the experimental products, this portion of the research allowed identification of AAVs with greatest abilities to compete in the presence of both other atoxigenic isolates and aflatoxin producers under field conditions. Similarly in Nigeria, one of the four constituent AAVs of the initial experimental product established poorly in field evaluations (Atehnkeng et al., 2014) and hence was not included as an active ingredient of the final multi-AAV biocontrol product Aflasafe^®^.

Bandyopadhyay et al. (2016) underscored the importance of distribution and incidence of AAVs with potential as aflatoxin biocontrol agents as proxies for adaptation, competitiveness, and fitness in target environments. However, superior adaptation should also reflect increased efficacy in the target crop (Mauro et al., 2015). We report substantial reductions in aflatoxin concentrations in both groundnut (70–100% less) and maize (50–100% less) from plots treated with mixtures of atoxigenic isolates belonging to genetically diverse AAVs across all three AEZs. Lower than expected aflatoxin levels were also detected in maize from control plots across AEZs and may reflect the effect of drift of atoxigenic fungi from treated plots to adjacent control plots due to the relatively short separation distance (5 m). Indeed, most AAVs of the applied isolates were detected in control crops (Table 7). Conidia of *A. flavus* are common constituents of air currents dispersed over short and long distances (Bennett, 2010). Thus, a distance of at least 500 m between a treated and a control plot is necessary to avoid inter-plot interference (Bock et al., 2004; Atehnkeng et al., 2014).

Atehnkeng et al. (2014) demonstrated that mixtures of atoxigenic isolates are effective at reducing aflatoxin contamination in maize. Our results suggest that atoxigenic isolates mixtures belonging to distinct AAVs can be strategically designed for aflatoxin reduction in both maize and groundnut cropping systems in Ghana. Eight atoxigenic isolates belonging to atoxigenic AAVs were selected as active ingredients of two biocontrol products for aflatoxin mitigation and subsequently registered with Ghana’s Environmental Protection Agency (Table 8). Six of the eight selected isolates had total or partial deletions in the cyclopiazonic acid (CPA) gene cluster while two produced undetectable amount of CPA (unpublished data). For the selection of the active ingredient AAVs, we considered their frequency of occurrence (Table 1), the competitive potential against aflatoxin producers (Table 3) and the relative adaptation in the evaluated maize and groundnut treated and control soils and crops (Table 7). This systematic evaluation protocol offered the opportunity to select the best possible combinations of active ingredients among the evaluated AAVs. However, all experimental products evaluated in the current study were efficient in reducing aflatoxin contamination of both maize and groundnut and each of the 12 AAVs were able to disperse to and increase frequency on the target crops. The selection strategy provides a basis for use of the most detected AAVs. However, even use of the most poorly adapted isolates examined here would provide better crop protection and increased food safety than failure to use atoxigenic strain-based biocontrol.

Application of atoxigenic *A. flavus* isolates on a target crop is a deliberate action to reshape fungal community composition in favor of the applied atoxigenic isolates due to founder events and competitive exclusion resulting in displacement of aflatoxin producers (Cotty and Bayman, 1993; Cotty et al., 2007; Mehl et al., 2012). Effective displacement of resident aflatoxin producers is achieved through proper timing of biocontrol applications during critical crop developmental stages (2–3 weeks before crop flowering) prior to the natural increase of the local *Aspergillus* population (Bandyopadhyay et al., 2016). Timed applications offer atoxigenic genotypes the advantage of becoming the founding population (Cotty and Mellon, 2006; Cotty et al., 2007) to quickly multiply and disperse to other nutrient sources and the target crop so that aflatoxin producers become less frequent (Bandyopadhyay et al., 2016).

In the current study, substantial displacement of aflatoxin producers from soils and crops occurred in treated plots across all three AEZs. The displacement was observed also in the non-treated crops. The *A. flavus* L morphotype largely dominated communities of *Aspergillus* section *Flavi* in soils collected before treatment, soils at harvest, and grains from both treated and control plots. The L morphotype is recognized as the most successful colonizer of soil and other substrates including grains in similar studies (Alanis Zanon et al., 2013; Atehnkeng et al., 2014; Doster et al., 2014). Frequencies of *A. parasiticus* were low (<1%), as reported previously in Ghana (Agbetiameh et al., 2018). Factors leading to low frequencies of this species in groundnut in West Africa remain unknown. Similarly, *A. parasiticus* is not common in portions of the Middle East (Lisker et al., 1993). In other regions of Southern Africa and North America, *A. parasiticus* is an important causal agent of groundnut aflatoxin contamination (Horn and Dorner, 1998; Kachapulula et al., 2017).

## Conclusion

Twelve atoxigenic African *A. flavus* vegetative compatibility groups (AAVs) commonly occurring across Ghana were characterized. The potential of a representative member of each AAV to inhibit aflatoxin contamination of maize grains was assessed in laboratory assays. AAV adaptation in maize and groundnut cropping systems in three AEZs in Ghana was assessed. The results formed the basis for selection of eight superior atoxigenic *A. flavus* isolates, each belonging to an unique AAV, as active ingredients of two biocontrol products, Aflasafe GH01 and Aflasafe GH02, for use on maize and groundnut in Ghana (Table 8). The unique SSR patterns of the eight atoxigenic isolates (Table 2) can serve as a resource for identification of the active ingredients of each of Aflasafe GH01 and Aflasafe GH02 after field application. Use of the identified atoxigenic AAVs offers a sustainable management option for aflatoxin mitigation in maize and groundnut for smallholder farmers in Ghana providing an inexpensive opportunity for improved food safety, productivity, and income.

## Data Availability

All datasets generated for this study are included in the manuscript and/or the Supplementary Files.

## Author Contributions

DA, JA, PC, and RB contributed to the conception and design of the experiments. DA and JA conducted the experiments and field studies, and collected and analyzed the data. AO-B, RA, PC, and RB provided the guidance. KC, M-SI, and PC conducted the molecular studies. DA, AO-B, KC, PC, and RB drafted the manuscript. All authors read, reviewed, and approved the final version of the manuscript. RB and AO-B secured funds for the study.

## Conflict of Interest Statement

The authors declare that the research was conducted in the absence of any commercial or financial relationships that could be construed as a potential conflict of interest.
