# Supplementary material for: Potential of Atoxigenic Aspergillus flavus Vegetative Compatibility Groups Associated With Maize and Groundnut in Ghana as Biocontrol Agents for Aflatoxin Management
Source: Front Microbiol. 2019 Sep 6;10:2069. doi: 10.3389/fmicb.2019.02069 (PMC6743268; doi:10.3389/fmicb.2019.02069)
Supplement: Supplementary file 4 [file Table_4.docx]

**Supplementary Table 4.** Incidence of isolates belonging to atoxigenic African *Aspergillus flavus* vegetative compatibility groups (AAVs) in groundnut kernels from plots treated with three experimental products and their corresponding controls in three agroecological zones (AEZs) in Ghana

| Products | AAV | Plot | AEZ^u^ | Region^v^ | District^w^ | Samples^x^ | Isolates^y^ | Rank^z^ |
| --- | --- | --- | --- | --- | --- | --- | --- | --- |
| A | GHG331-8 | Treated | 3 | 4 | 6 | 9 | 17 | 8 |
|  |  | Control | 1 | 1 | 1 | 1 | 1 | 8 |
|  | GHG079-4 | Treated | 2 | 3 | 3 | 4 | 6 | 10 |
|  |  | Control | 0 | 0 | 0 | 0 | 0 | 9 |
|  | GHM109-4 | Treated | 3 | 5 | 7 | 9 | 24 | 5 |
|  |  | Control | 3 | 4 | 4 | 5 | 6 | 2 |
|  | GHM174-1 | Treated | 3 | 4 | 8 | 11 | 32 | 6 |
|  |  | Control | 2 | 4 | 4 | 4 | 4 | 5 |
| B | GHM173-6 | Treated | 0 | 0 | 0 | 0 | 0 | 11 |
|  |  | Control | 0 | 0 | 0 | 0 | 0 | 9 |
|  | GHG083-4 | Treated | 3 | 5 | 10 | 15 | 52 | 1 |
|  |  | Control | 2 | 3 | 4 | 7 | 13 | 6 |
|  | GHM287-10 | Treated | 3 | 5 | 8 | 16 | 27 | 2 |
|  |  | Control | 2 | 2 | 3 | 4 | 4 | 7 |
| C | GHM017-6 | Treated | 2 | 3 | 4 | 6 | 14 | 9 |
|  |  | Control | 0 | 0 | 0 | 0 | 0 | 9 |
|  | GHM511-3 | Treated | 3 | 4 | 8 | 14 | 52 | 4 |
|  |  | Control | 3 | 4 | 5 | 8 | 10 | 1 |
|  | GHG321-2 | Treated | 3 | 5 | 6 | 9 | 18 | 7 |
|  |  | Control | 3 | 3 | 4 | 4 | 7 | 3 |
|  | GHM001-5 | Treated | 3 | 5 | 8 | 13 | 29 | 3 |
|  |  | Control | 3 | 3 | 3 | 3 | 9 | 4 |

^u^ Number of AEZs out of three from which an applied AAV was isolated.

^v^ Number of regions out of five from which an applied AAV was isolated.

^w^ Number of districts out of 10 from which an applied AAV was isolated.

^x^ Number of samples out of 30 from which an applied AAV was isolated.

^y^ Number of isolates out of 360 belonging to the applied AAV.

^z^ Rank of the applied AAV based on its recovery in diverse AEZs, regions, districts and number of samples from which it was isolated. To calculate the rank, the proportion of the number of i) AEZ (n = 3), ii) regions (n = 5), iii) districts (n = 10), and iv) samples (n = 30) where the AAV was detected and v) the proportion of isolates of the AAV detected (n = 360) was summed. Higher the sum, higher (1 = highest, 11 = lowest) the rank.
